# Conservative Management of Pneumoperitoneum in Necrotising Enterocolitis- Is it Possible?

**Published:** 2016-04-10

**Authors:** Anand Pandey, Shailendra P Singh, Vipin Gupta, Rajesh Verma

**Affiliations:** 1 Department of Pediatric Surgery King George's Medical University, Lucknow, India; 2 Department of Surgery, UP Rural Institute of Medical Sciences and Research, Saifai, Etawah, 206130, India

**Keywords:** Necrotising enterocolitis, Pneumoperitoneum, Neonates, Premature, Peritoneal tapping

## Abstract

**Introduction:** Necrotizing enterocolitis (NEC) is a common in neonatal intensive care unit (NICU) patients; especially in premature and low birth weight ones. Surgery is indicated when there is pneumoperitoneum. Other therapies include conservative observation or primary peritoneal drain (PPD). This study was conceived to evaluate peritoneal tapping, rather than primary peritoneal drain (PPD) as a treatment of NEC.

**Material and Methods**: This prospective observational study conducted from December 2012 to December 2014 and including all patients of NEC having pneumoperitoneum on X-ray.

**Results**: There were 12 patients of NEC. Seven patients responded to single peritoneal tapping. Three patients needed one more tapping. Laparotomy was required in remaining two patients. One patient, who underwent laparotomy, expired due to severe sepsis. The mean duration of follow up was 4.83 months (range 2 to 8).

**Conclusion**: Peritoneal tapping in NEC who develops pneumoperitoneum appears to be a viable option. Further studies in this regard may substantiate this mode of therapy.

## INTRODUCTION

NEC is common in NICU patients especially premature and LBW babies following initiation of enteral feeding [1] and cause of significant morbidity and mortality [2,3]. NEC is classified according to Bell’s staging a stage I (suspect), stage II (definite), or stage III- (advanced disease) [4]. Surgery has been the standard for managing the pneumoperitoneum.

Non-surgical management of this entity in form of peritoneal tapping has been reported sporadically [5]. This study was undertaken to see if peritoneal tapping is a viable treatment option in patients of pneumoperitoneum due to NEC.

## MATERIALS AND METHODS

This study was conducted from December 2012 to December 2014. All patients of NEC having pneumoperitoneum on X-ray were included in this study. Patient were admitted directly or referred from the Paediatrics department. All patients were put on intravenous (IV) antibiotics covering aerobic and anaerobic organisms. Clinical parameters such as pulse rate, respiratory rate, temperature, and capillary filling time (CFT) were monitored. Complete blood counts, renal function test, electrolytes, and C reactive protein (CRP) were recorded. An assessment of abdominal girth at umbilical level and abdominal wall erythema was made, both before and after the peritoneal tapping.

Under aseptic precautions, a 22 Fr IV cannula was inserted into the peritoneal cavity in the epigastric region about 1cm to the left of midline. Right side was avoided to prevent inadvertent injury to the umbilical vein. The peritoneal cavity was deflated with the help of cannula, which was left at its place for about two hours in a sterile close circuit. If there was recurrence of abdominal distension after more than 24 hours of first tapping, a repeat peritoneal tapping was performed. After two attempts of peritoneal tapping, if there was recurrence of distension, laparotomy was performed within 24 hours. 

A positive outcome was made on the basis of decreased abdominal girth, improvement in abdominal wall erythema, improved values of blood count, renal function tests, and CRP. The patients were allowed orally on 7th day after peritoneal tapping. At that time, oral probiotics were also started. They were discharged after 14 days of IV antibiotics.

## RESULTS

There were 12 patients of NEC with pneumoperitoneum (table 1). Of these, five were premature and seven were full term neonates. Male to female ratio was 2:1. The mean birth weight of the patients was 2108.33 grams (range 1700 to 2800). At the time of presentation, the mean pulse rate and respiratory rate were 109.83 (range 95 to 120) and 55.75 (range 50 to 62) respectively. The mean total leukocyte count was 10083.33 (range 3000 to 18000). 

**Figure:**
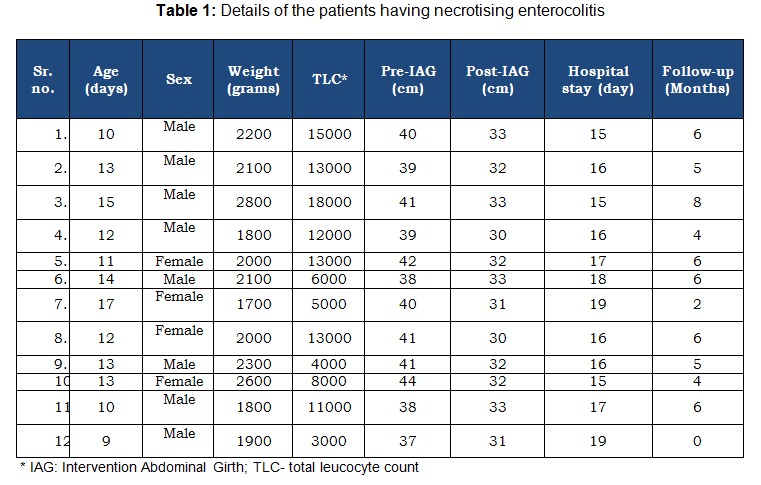
Table 1: Details of the patients having necrotising enterocolitis

At the time of peritoneal tapping, the mean abdominal girth was 40 cm (range 37 to 42). After abdominal decompression, it was 31.83 (range 30 to 33). Seven patients responded to single peritoneal tapping. Of these, six were full term and one was premature neonate. Three patients needed one more tapping. Of these, one was full term and remaining two were premature neonates. Laparotomy was required in remaining two premature patients, which revealed extensive disease. One patient, who underwent laparotomy, expired due to severe sepsis. 

The mean duration of hospital stay was 16.58 days (range 15 to 19 days). At the time of discharge, all patients had normal vitals and serum biochemistry. The mean duration of follow up was 4.83 months (range 2 to 8). There was no problem in the follow up of all patients.

## DISCUSSION

Mild cases of NEC may be managed by medical management [3] which includes cessation of enteral feeding, empiric antibiotics, and supportive care [2, 6]. The available surgical options include laparotomy or primary peritoneal drainage (PPD). PPD has been evaluated in various studies, some claiming it to be useful while others found it doubtful [7-10]. Two randomized control trials [11, 12] have found that survival rates were not statistically different between peritoneal drainage and laparotomy groups. A recent Cochrane Review, combining the results of the two studies, concluded that no significant benefits or harms of peritoneal drainage versus laparotomy could be identified [13].

The success of peritoneal tapping in this study was about 83%. The technique of peritoneal tapping differs from that of PPD, which involves penrose drain placement under vision. Peritoneal tapping is a less invasive method, whereas PPD involves opening of the peritoneal cavity and placement of the drain. Hence, we feel that is not justified to place peritoneal tapping at par with PPD as regard to invasiveness. Despite the claims that pneumoperitoneum denotes extensive disease, which requires immediate surgery, we feel that many such patients may have small perforations in the inflamed bowel. When supported with appropriate supportive management and peritoneal decompression, the bowel gets a chance to heal. However, we do agree that this is a speculation, as those patients who responded to the treatment were not explored surgically so as to look at the bowel condition; but since these patients responded, we assume it to be true. The tapping was successful in both premature and mature neonates; however, given the limited number of patients, we did not apply any statistical test between them. This may be considered as a limitation of this study. Also, it may be queried that why NEC occurred in full term neonates. In this regard, it is imperative to see that most of our patients, despite being full term, were low birth babies (table 1). Low birth is also a risk factor for NEC [3].

## CONCLUSION

To conclude, conservative management of pneumoperitoneum by peritoneal tapping in NEC appears to be a viable option. Further studies in this regard may substantiate our efforts. 

## Footnotes

**Source of Support:** None

**Conflict of Interest:** None
